# Nonobstructive hydronephrosis due to social polydipsia: a case report

**DOI:** 10.1186/1752-1947-6-376

**Published:** 2012-11-06

**Authors:** Natallia Maroz, Uladzimir Maroz, Saima Iqbal, Ravi Aiyer, Ganesh Kambhampati, A Ahsan Ejaz

**Affiliations:** 1 Division of Nephrology, Hypertension, and Renal Transplantation, University of Florida, 1600 Archer Road, PO Box 100224, Gainesville, FL, 32610, USA

## Abstract

**Introduction:**

Excessive fluid intake can lead to water intoxication, electrolyte abnormalities, exacerbation of heart failure and anatomical changes in the urinary tract that may present diagnostic and therapeutic challenges for patients and physicians. Although the development of nonobstructive hydronephrosis is recognized in patients with central and nephrogenic diabetes insipidus, pregnancy or psychiatric polydipsia, it is rarely a diagnostic consideration in healthy individuals with excessive fluid ingestion. We now present what we believe to be the first report of nonobstructive hydronephrosis associated with social polydipsia.

**Case presentation:**

A 53-year-old African-American woman with moderate back pain was found to have bilateral moderate hydronephrosis and hydroureter by abdominal computed tomography. She underwent ureteral stent placement followed by exploratory laparoscopy with lysis of adhesions and a right oophorectomy, without resolution of the nonobstructive hydronephrosis. A careful assessment revealed a social habit of consuming approximately 5.5L of fluid daily in an effort to remain hydrated in accordance with public health service announcements. It was recommended that the patient reduce her fluid intake. A repeat ultrasound after six weeks revealed complete resolution of the bilateral hydronephrosis and hydroureter.

**Conclusion:**

Recognition of the nonobstructive nature of hydronephrosis caused by polydipsia in healthy individuals is important to prevent unnecessary interventions.

## Introduction

In 1945, the United States Food and Nutrition Board of the National Research Council published dietary recommendations specifying a daily water intake of 2.5 L [1], concluding that the majority of this quantity is contained in prepared foods. Despite incomplete evidence for the recommendations and general ignorance of the presence of dietary water in prepared foods, the guidelines have prompted six decades of popularizing ‘eight 8-ounce glasses of water per day’ for health maintenance. These recommendations were amended in collaboration with Health Canada in 2004 with suggested requirements to consume 2.7L of fluid per day for women and 3.7L for men with an upper level for water consumption [2]. The ‘stay hydrated’ health maintenance concept has encouraged people to consume fluids, often in excess of their natural thirst, and sometimes led to water-balance abnormalities such as life-threatening hyponatremia, respiratory failure and decompensation of heart failure in patients with comorbid conditions. Even healthy athletes have been reported to develop serious health complications with only moderately excess fluid intakes [3]. Although the prevalence of polydipsia ranges from 6% to 17% among chronically ill psychiatric patients [4], the full extent and range of adverse events associated with excessive fluid intake remain uncertain in healthy populations. We highlight these issues using an unusual case of nonobstructive hydronephrosis (NOH) associated with social polydipsia.

## Case presentation

**Figure 1:**
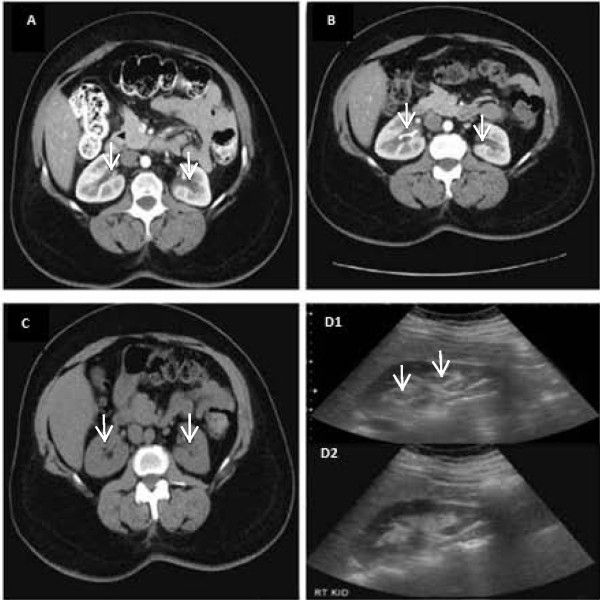
**Time course of hydronephrosis. **(**A**) Abdominal computed tomography. Initial presentation; mild to moderate bilateral hydronephrosis. (**B**) Abdominal computed tomography. Persistent hydronephrosis despite ureteral stent placement in right kidney. (**C**) Abdominal computed tomography. Persistent hydronephrosis after exploratory laparoscopy and right oophorectomy. (**D**) Ultrasound of right kidney. (D1) Mild hydronephrosis prior to reduction of fluid intake. (D2) Resolution of hydronephrosis after fluid intake reduction.

A 53-year-old African-American female medical assistant with a past medical history of asthma, diabetes mellitus for two years and a hysterectomy was referred to our nephrology clinic for a second opinion regarding persistent bilateral hydronephrosis. Her family history did not include nephrogenic or central diabetes mellitus or malignancies. Twelve months prior to the renal consultation, our patient presented to the emergency department with mild-to-moderate bilateral flank pain without fever, chills, dysuria, difficulty urinating or hematuria. Her blood and urine chemistry and a complete blood count tests yielded normal results (Table 1). Abdominal computed tomography (CT) revealed the presence of moderate bilateral hydronephrosis and hydroureter (Figure 1A). Given the normal laboratory findings for renal function and good urine output at home and in the emergency department, she was discharged with symptomatic treatment and a referral to a local urologist for further evaluation. Her only prescription medication was 50mg of sitagliptin daily.

**Table 1:** Laboratory tests

**Tests**	**Emergency department**	**Second opinion**	**Follow-up**
	**Time 0 months**	**Time 8 months**	**Time 10 months**
**Serum**			
Sodium (meq/L)	137	139	140
Potassium (meq/L)	4.3	4.1	4.3
Chloride (meq/L)	101	102	102
Bicarbonate (meq/L)	25	26	27
Blood urea nitrogen (mg/dL)	11	12	11
Creatinine (mg/dL)	0.91	0.93	0.86
Glucose (mg/dL)	103	127	104
Osmolality (mOsm/kg)		286	294
White blood count (×1000/mm^3^)	5.9	5.2	4.8
Hemoglobin (gm/dL)	13.9	14.7	14.9
Hematocrit (%)	43.5	46.1	43.8
Platelet (×1000/mm^3^)	196	184	185
**Urine**			
Specific gravity	1.008	1.009	1.023
pH	6.5	6.5	6.2
White blood cells (/hpf)	0 to 5	0 to 5	0 to 5
Red blood cells (/hpf)	0	0	0
Random protein (mg)		66	39
Random creatinine (mg)		118	75
Osmolality (mOsm/kg)		334	502

*hpf* high power field.

After a delay of six months for health insurance issues, a repeat CT and intravenous pyelogram were performed by our patient’s urologist that confirmed persistent, moderate hydronephrosis on her right side, and mild hydronephrosis on her left side. Despite a lack of any anatomical abnormality evident by cystoscopy, a right ureteral stent was inserted (Figure 1B). The discomfort in our patient’s flank pain persisted; a repeat abdominal CT performed two months later did not show resolution or improvement of the hydronephrosis, and the ureteral stent was removed. She was then evaluated by a gynecologist and underwent exploratory laparoscopy with lysis of adhesions and a right oophorectomy. Another abdominal CT, performed a month after the exploratory laparoscopy, failed to show any improvement in the degree of bilateral hydronephrosis (Figure 1C). Our patient was then referred to an academic medical center for a second opinion regarding persistent bilateral hydronephrosis.

Our patient complained only of intermittent, mild, bilateral flank pain that was unrelated to physical activity, but sometimes related to fluid intake. She reported drinking 4.5L to 5.5L of fluid daily for the last three years, stating that ‘all (her) friends do so to stay healthy.’ Her physical examination revealed the following: blood pressure, 136/90mmHg; heart rate, 87 beats per minute; temperature, 36.9°C; height, 165cm; weight, 8.7kg; body mass index, 29.61kg/m^2^; normal heart and lung examination results; no organomegaly or tenderness on abdominal examination; no suprapubic fullness; mild right costovertebral angle tenderness with percussion; no extremity edema; and normal musculoskeletal and neurological examination results. Her laboratory data is presented in Table 1. A renal ultrasound revealed bilateral moderate hydronephrosis with normal echogenicity of the parenchyma (Figure 1D1).

Based on her history, her laboratory and imaging study results, and previous evaluation, our patient was suspected to have nonobstructive fullness in the urine excretory system as a result of a mismatch of its capacity to produce excessive urine volume. She was advised to decrease her fluid intake to less than 2L/day. A mercaptoacetyltriglycine (MAG-3) nuclear renogram with furosemide (Lasix) showed prompt bilateral excretion with no evidence of any obstruction. A repeat renal ultrasonography after six weeks showed normal-sized kidneys and complete resolution of the hydronephrosis (Figure 1D2). Our patient was discharged from the nephrology clinic with recommendations to drink according to her thirst and follow-up with her local physician.

## Discussion

NOH is an uncommon entity that occurs in the pediatric and adult populations, primarily in patients with central and nephrogenic diabetes insipidus, both sporadically and in familial clusters [5]. NOH has been reported in 67% of patients with nephrogenic diabetes insipidus [6] and is not uncommon in those with psychogenic polydipsia [7]. In mice, mutations in the vasopressin-neurophysin II gene have been associated with familial neurohypophyseal diabetes insipidus [8], and the S256L mutation in aquaporin-2 has been associated with congenital progressive hydronephrosis [9]. Our patient’s condition differs from that in other published reports in that the NOH was not caused by psychogenic polydipsia, diabetes insipidus, obstruction, or genetic abnormalities, but was a sequela of an exaggerated social habit.

The lack of electrolyte abnormalities in our patient was remarkable. In fact, the ability to maintain a stable serum sodium level and concentrate urine with water deprivation along with bilateral hydronephrosis-hydroureter in the absence of an obstruction is diagnostic for NOH. The long-term consequences of NOH are not known, with reports of partial or complete resolution of hydronephrosis in the majority of reports. Several cases of familial nephrogenic diabetes insipidus were followed over periods of decades with observation of NOH, but no harmful impact on renal function was observed. A few cases have been described of reversible renal impairment in patients with NOH due to bladder overdistension and delayed voiding [10,11]. Rupture of the urinary tract and progression to end-stage kidney disease were also reported [12,13]. It is important to be cognizant of the latest outcome data from acute kidney injury research, which demonstrates a high risk of developing chronic kidney disease and the need for renal replacement therapy in patients with even mild increments in serum creatinine [14].

The average capacity of the urinary system in a nonobstructive state for urine volume in an adult patient is 600mL to 1000mL with a bladder capacity of 400ml to 800mL. The average healthy adult perceives the need to void with a bladder filled to 50% of its capacity. Employed individuals frequently restrict their use of the bathroom during working hours, thereby promoting dilation of the urinary system. NOH represents benign fullness in the urine excretory system as a result of a mismatch of its capacity with respect to the excessive production of urine. The persistently large urine volumes in NOH lead to urinary bladder distension and hypertrophy, with subsequent intramural obstruction of the distal ureters. In time, bladder contractility is compromised, ureteric peristalsis diminishes, and large residual urine volumes worsen this functionally obstructive uropathy. Other studies have reported that normal detrusor contractility is maintained in NOH even with a large postvoidal urine residue and the dilation of the bilateral renal pelvis, ureter and bladder [15].

NOH is a well-recognized entity during pregnancy and can be seen in up to 80% of pregnant women, with more prominent occurrence on the right side. The renal pelvis and calyces become dilated in pregnancy as a result of progesterone effects. It is important to emphasize that hydronephrosis caused by obstruction is much more common than NOH and frequently presents with flank pain and worsening of renal function. Although our patient had mild flank pain at presentation, her urological work-up results did not reveal the presence of ureteropelvic junction obstruction or vesicoureteral reflux, which would be suggestive of anatomical abnormalities. Patients with polydipsia can potentially develop a variant of a functional ureteropelvic junction obstruction because of the massive volume of urine exceeding the drainage capacity of the renal pelvis. It is important to recognize the presence of bilateral hydronephrosis and hydroureter (primarily in the upper and middle segments) in cases of NOH.

## Conclusion

Fluid intake is beneficial in the prevention and management of nephrolithiasis, as well as weight loss. It was shown to suppress plasma levels of arginine vasopressin in animal models of polycystic kidney disease, thereby slowing cyst progression. Fluid intake also improves glomerular filtration rate and protects against the progression of chronic kidney disease. Unfortunately, there are many clinical scenarios in which the excessive consumption of water can lead to worsening of electrolyte abnormalities and decompensation in patients with heart, renal and liver failure - demonstrating that people with underlying comorbidities need to follow the specific recommendations of medical professionals. Patients may experience complications from social polydipsia, under the influence of the urban myth of consuming eight 8-ounce glasses of water per day for good health. Our patient developed NOH as a result of social polydipsia, the etiology of which went unrecognized and led to unnecessary, risky and expensive interventions. This may be an example of exaggerated social habits promoted by modern media, a peril of practicing medicine in 21st century.

## Consent

Written informed consent was obtained from the patient for the publication of this case report and accompanying images. A copy of the written consent is available for review by the Editor-in-Chief of this journal.

## Competing interests

The authors declare that they have no competing interests.

## Authors’ contributions

NM was a major contributor in writing the manuscript and was involved in bibliographic research. UM was involved in bibliographic research and the interpretation of images. SI was involved in the clinical care of the patient and was involved in the bibliographic research. RA was involved in bibliographic research and obtaining medical records. GK was involved in the bibliographic research. AAE was a major contributor in writing the manuscript. All authors have read and approved the final manuscript.

## References

[B1] Food and Nutrition Board, National Academy of Sciences Recommended dietary allowances Natl Res Coun, Repr and Circ Ser 1945 122 3 18

[B2] Institute of Medicine (U.S.) Food and Nutrition Board Panel on Dietary Reference Intakes for Electrolytes and Water, Standing Committee on the Scientific Evaluation of Dietary Reference Intakes: Dietary reference intakes: water, potassium, sodium, chloride and sulfate 2004 Washington DC: National Academies Press

[B3] Shapiro SA Ejaz AA Osborne MD Taylor WC Moderate exercise-induced hyponatremia Clin J Sport Med 2006 16 72 73 10.1097/01.jsm.0000188042.04760.09 16377980

[B4] Illowsky BP Kirch DG Polydipsia and hyponatremia in psychotic patients Am J Psychiatry 1988 145 675 683 3285701 10.1176/ajp.145.6.675

[B5] Urribari J Kaskas M Hereditary nephrogenic diabetis insipidus and bilateral non-obstructive hydronephrosis Nephron 1993 65 346 349 10.1159/000187510 8289981

[B6] Nakada T Miyauchi T Sumiya H Shimazaki J Nonobstructive urinary tract dilatation in nephrogenic diabetes insipidus Int Urol Nephrol 1990 22 419 427 10.1007/BF02549772 2076930

[B7] Blum A Friedland GW Urinary tract abnormalities due to chronic psychogenic polydipsia Am J Psychiatry 1983 140 915 916 6859314 10.1176/ajp.140.7.915

[B8] Miyakoshi M Kamoi K Murase T Sugimura Y Oiso Y Novel mutant vasopressin-neurophysin II gene associated with familial neurohypophyseal diabetes insipidus Endocr J 2004 51 551 556 10.1507/endocrj.51.551 15644573

[B9] McDill BW Li SZ Kovach PA Ding L Chen F Congenital progressive hydronephrosis (cph) is caused by an S256L mutation in aquaporin-2 that affects its phosphorylation and apical membrane accumulation Proc Natl Acad Sci USA 2006 103 6952 6957 10.1073/pnas.0602087103 16641094 PMC1459000

[B10] Streitz JM Jr Streitz JM Polyuric urinary tract dilation with renal damage J Urol 1988 139 784 785 3352042 10.1016/s0022-5347(17)42636-x

[B11] Ulinski T Grapin C Forin V Vargas-Poussou R Deschênes G Bensman A Severe bladder dysfunction in a family with ADH receptor gene mutation responsible for X-linked nephrogenic diabetes insipidus Nephrol Dial Transplant 2004 19 2928 2929 15496575 10.1093/ndt/gfh486

[B12] Soler Fernández JM Caravaca Magariños F Domínguez Bravo C Murillo Mirat J Herrera Puerto J Sanz Cueva J Polyuric dilatation of the urinary tract in congenital nephrogenic diabetes insipidus. Clinical and diagnostic aspects. Presentation of a case and review of the literature Actas Urol Esp 1990 14 374 377 2288259

[B13] Van Lieburg AF Knoers NV Monnens LA Clinical presentation and follow-up of 30 patients with congenital nephrogenic diabetes insipidus J Am Soc Nephrol 1999 10 1958 1964 10477148 10.1681/ASN.V1091958

[B14] Coca SG Peixoto AJ Garg AX Krumholz HM Parikh CR The prognostic importance of a small acute decrement in kidney function in hospitalized patients: a systematic review and meta-analysis Am J Kidney Dis 2007 50 712 720 10.1053/j.ajkd.2007.07.018 17954284

[B15] Jin XD Chen ZD Cai SL Chen SW Nephrogenic diabetes insipidus with dilatation of bilateral renal pelvis, ureter and bladder Scand J Urol Nephrol 2009 43 73 75 10.1080/00365590802580208 19037828

